# Does Obstructive Sleep Apnea Syndrome Have Negative Effects on Hearing?

**DOI:** 10.22038/IJORL.2022.64912.3225

**Published:** 2023-01

**Authors:** Fevzi Solmaz, Buse Ekim, Abdullah Simsek

**Affiliations:** 1 *Department of Otorhinolaryngology, Health Sciences University Bursa Training and Research Hospital, Bursa, Turkey.*; 2 *Department of Chest Diseases, Health Sciences University Bursa Training and Research Hospital, Bursa, Turkey.*

**Keywords:** Audiometry, Hearing, Obstructive Sleep Apnea Syndrome

## Abstract

**Introduction:**

The our aim was to research the occurrence of hearing loss associated with the effect of hypoxemia on inner ear structures owing to sleep apnea syndrome and to designate the timely signs of cochlear injury.

**Materials and Methods:**

Participants diagnosed with probable sleep-disordered breathing among 63 patients, who experienced polysomnographic examination, were unexcluded in the present study. Control and study groups were structured in four groups pursuant to the apnea-hypopnea index and an intergroup comparison of audiometric parameters was performed. Accordingly, the apnea-hypopnea index, speech discrimination scores, speech recognition thresholds, and pure tone thresholds were compared.

**Results:**

A comparison of the obstructive sleep apnea groups by the degree of hearing loss indicated that there were significant differences by the average pure tone audiometry, average speech recognition thresholds, and average speech discrimination scores in both ears between the four groups (p<0.001).

**Conclusion:**

The results of the study proposed that intermittent hypoxemia due to obstructive sleep apnea syndrome might have adverse effects on both the speech discrimination and hearing.

## Introduction

Obstructive sleep apnea syndrome is a sleep-relevant disturbance characterised by episodes in patients with extended complete (apnea) or/and partial (hypopnea) obstruction of upper airway. 2% of women and 4% of men have been affected by these conditions (1). Decreased blood oxygen saturation induces intermittent hypoxia and hypercapnia with elevated carbon dioxide concentration levels.

Hypoxemia is interrelated meaningful adverse effects on hemodynamic and biochemical regulatory mechanisms in the peripheral and central nervous systems. 

Associated with chronic hypoxemia, a series of pathological events can be triggered by neurocircuitry dysfunctions and decreased levels of adenosine (2-4). Furthermore, it is established that recurrent stress secondary to chronic intermittent hypoxia is related to endothelial disfunction in the microcirculation. It was suggested that the foregoing condition impaired the vascular supply of peripheral nerves and induced loss in neural function (1,5).

The hypoxia and normoxia episodes were reported as the basic factors affecting the fabrication of reactive oxygen derivatives and triggering oxidative stress and endothelial irregularity (1). 

From the cochlea through the auditory pathways to the auditory center in the cortex, the inner ear structures are the mechanisms that are vulnerable to the effects of hypoxemia (6). OSAS diagnosis is considered an independent predictor for cardiovascular disorders, including stroke, coronary artery disease, and hypertension (7-9). 

The our study intented to research the effect of hypoxemia associated with OSAS on inner ear structures and to detect the early signs of cochlear damage. The results of the study were discussed in the light of the relevant literature on the auditory functions of patients with OSAS.

## Materials and Methods

The study was performed in a tertiary hospital.

Participants were wanted to complete in an informed consent form and the relevant approval was gained from the ethics committee (Protocol No: 2011-KAEK-25 2020/11-11). Participants diagnosed with probable sleep-related breathing disorder among 63 patients, who experienced polysomnographic examination, were unexcluded in the study.

The mean ages of patients in the OSAS groups are presented in Table 1. The gender distribution by OSAS groups is presented in Table 2. A review of Table 2 suggested that the gender distributions in the groups was similar (p=0.209). Polysomnography tests were performed by the department of pulmonary medicine in the sleep laboratory at the same institute.

All the participants underwent an otolaryngologic examination by a senior otolaryngologist. Patients with obstructive pulmonary disease, hypertension and diabetes mellitus associated with hypoxemia and microvascular circulatory disorders, including patients with an acute or chronic otolaryngologic disease and chronic systemic diseases and patients with history of hearing loss diseases, such as chronic otitis media, or SNHL were excluded from the study. 

The group of study was composed of three sub-groups of Mild-1, Moderate-2, and Severe-3, rely on the severity of the illness. We have recorded index of apnea hypopnea and blood oxygen saturation values of the patients, and have mesured the blood oxygen saturation levels using a pulse-oximeter. Simple snoring, in the absence of obstructive sleep apnoea, is a common problem. The control group consisted of individuals with an apnea hypopnea index level of <5. 

An apnea hypopnea index of less than 5 is considered normal (simple snoring group). The study group further divided into three sub-groups including patients with Mild (index: 5-15, blood oxygen saturation >85%), Moderate (index:15-30, blood oxygen saturation =65-84%), and Severe (index:>30, blood oxygen saturation <65%) OSAS. Participants’ body mass index (BMI) could not have been examined and therefore excluded from analysis. 

*Polysomnography Test*

Sleep studies patients experienced overnight Polysomnography carried out by study-confirmed technicians using a standardised protocol and in accordance with the guidelines of the American Academy of Sleep Medicine (10). 

We have scored sleep stages as regards standard criteria in 30-second periods. We have performed overnight polysomnography using the 16-channel system of Embla (Iceland). 

The system comprises of 4 channels of electroencephalography and 2 channels of EOG, recording submental EMG, electrocardiogram readings, tracheal sound, body position, oronasal airflow, tibial EMG, abdominal and thoracic movements, oxygen saturation. We evaluated the parameters of PSG through a variety of measurements and indices: respiratory disturbance index, apnea-hypopnea index; oxyhemoglobin saturation (%); number of respiration events including numbers of apnea or hypopnea events; snoring index; heart rate (pulses/min).

Apnea was recognised as whole pause of airflow for more than 10 seconds. Hypopnea was identified as >30% airflow reduction lasting longer than 10 seconds with >4% desaturation and/or arousal. The mean count of apnea and hypopnea episodes per hour of sleep was measured as apnea-hypopnea index. Apnea-hypopnea index was measured according to the recommendations of the American Academy (10).

Patients were diagnosticated with OSA if their apnea-hypopnea index was ≥5. To rate the difficulty of sleep apnea, the number of events per hour is reported as the apnea-hypopnea index. A normal is considered apnea-hypopnea index of less than five. An apnea-hypopnea index of more than 30 events severe, 15-30 moderate and 5-15 is mild per hour characterize sleep apnea.

*Audiometry Tests*

Tympanometry test (TT), speech discrimination (SD), speech receiving threshold (SRT) and pure tone audiometry tests (PTA-Air/Bone) were applied to all groups. Pure tone audiometry test was applied using a clinical type device with dual channel. The participants wore headphones in a quiet booth, and hearing thresholds were designated at frequencies of 250-8000 Hz with the “decreasing” method and at frequencies of 500-4000 Hz with a bone vibrator. Pure tone average values were designated by obtaining the average of 500-4000 Hz frequencies. Tympanometric evaluation was performed using a 226 Hz probe tone stimulus with an intensity of 80 dB. Ipsilateral acoustic reflex was determined at frequencies of 500-4000 Hz with an intensity of 100 dB. 

*Statistical Analysis*

Based on the acquired data, the descriptive statistics were provided in the forms of percentage frequencies, number, quartiles (25th, median and 75th), and mean standard deviation (SD) based on the type of the features. Normal distribution hypothesis for numerical features were tested using the Kolmogorov-Smirnov test. The distribution of gender in the OSAS groups that were constituted in the light of the PSG results was compared with the Pearson chi-square test, where the mean age was compared with the one-way ANOVA model. Furthermore, the Fisher-Freeman-Halton exact test was used to analyze the intergroup differences in the Pure Tone Average (PTA)-Air and PTA-Bone grades. The ANCOVA model was used for the purposes of comparing the groups by hearing capacity (hearing loss). 

There was a significant intergroup difference by age, and therefore the ages were incorporated as covariates into the ANCOVA model with an aim to eliminate the effects of the age differences. 

The Post-Hoc Tukey test was performed to analyze the significant intergroup differences. We have considered a p value of <0.05 as statistically significant. SPSS 22 Version software package was performed for the statistical analyses of the study data.

## Results

The mean ages of participants in the OSAS groups are submitted in Table 1. 

**Table 1:** Mean age in OSAS groups

**Age**	**N**	**Mean**	**SD**	**Minimum**	**Maximum**
Simple Snoring	20	46,10	6,851	34	56
Mild OSAS	6	51,33	7,202	42	62
Moderate OSAS	10	51,20	8,066	32	60
Severe OSAS	27	56,37	5,464	39	65
Total	63	51,81	7,758	32	65

*Kolmogorov-Smirnov test, one-way ANOVA model.*

A comparison indicated that the mean age of individuals with simple-snoring was significantly lower compared to the severe-OSAS group, a comparison indicated that no significant difference among the groups (p >0.05).The gender distribution by OSAS groups is presented in Table 2. 

 A review of Table 2 suggested that we have not found significant difference among the groups (p=0.209). Presented in Table 3. 

**Table 2:** Gender distribution in OSAS groups

		**PSG**								**Total**
		**Simple Snoring**		**Mild OSAS**		**Moderate OSAS**		**Severe OSAS**		
		n	%	n	%	n	%	n	%	n
Sex	M	10	50,0	3	50,0	8	80,0	20	74,1	41
	F	10	50,0	3	50,0	2	20,0	7	25,9	22
Total		20		6		10		27		63

*Pearson chi-square test*

**Table 3:** Results of a comparison of OSAS groups by the degree of hearing loss:

	**PSG**									
	**Simple Snoring (n=20)**		**Mild OSAS (n=6)**		**Moderate OSAS (n=10)**		**Severe OSAS (n=27)**			
	n	%	n	%	n	%	n	%	Total n	**P**
PTA-Air-Right	20_a_	100,0	4_c_	66,7	10_a_	100,0	9_b_	33,3	43	<0.001
	0_a_	0,0	2_c_	33,3	0_a_	0,0	18_b_	66,7	20	
PTA-Bone-Right	20	100,0	6	100,0	10	100,0	23	85,2	59	0.231
	0	0,0	0	0,0	0	0,0	4	14,8	4	
PTA-Air-Left	20_a_	100,0	4_c_	66,7	8_c_	80,0	7_b_	25,9	39	<0.001
	0_a_	0,0	2_c_	33,3	2_c_	20,0	20_b_	74,1	24	
PTA-Bone-Left	20	100,0	5	83,3	9	90,0	24	88,9	58	0.274
	0	0,0	1	16,7	1	10,0	3	11,1	5	

^a^ *Fisher-Freeman-Halton exact test.* ^b^ *ANCOVA model.* ^c^ *Post-Hoc Tukey test.*

*A review of* Table 3 *suggested that*

There was no significant intergroup difference by PTA-Bone-Right and PTA-Bone-Left results, and that participants with a PTA-Air-Right hearing loss were mostly from the severe-OSAS group, followed by the mild-OSAS group. There was no participant with a PTA-Air-Right hearing loss in the other 2 groups. A PTA-Air-Left hearing loss was the most prevalent in the severe-OSAS group, followed by the mild-OSAS and moderate-OSAS groups. This result was not seen in the simple-snoring group at all.The results of a comparison of OSAS groups by the hearing capacity (hearing losses) are presented in Table 4. 

**Table 4:** Results of a comparison of OSAS groups by hearing capacities (hearing losses):

	**PSG**	**N**	**Mean**	**SD**	**Percentiles**			**P**
					**25**	**Median**	**75**	
SD-Right	Simple Snoring	20	98,50^a^	1,792	98,00	99,00	100,00	<0.001
	Mild OSAS	6	92,50^b^	5,244	88,75	92,50	96,25	
	Moderate OSAS	10	90,70^b^	3,831	88,00	90,00	93,00	
	Ağır OSAS	27	87,22^c^	4,925	85,00	88,00	90,00	
SD-Left	Simple Snoring	20	98,50^a^	2,565	98,00	100,00	100,00	<0.001
	Mild OSAS	6	91,33^b^	3,204	88,00	91,00	95,00	
	Moderate OSAS	10	91,90^b^	4,654	88,75	93,00	95,25	
	Severe OSAS	27	88,33^b^	4,739	85,00	88,00	92,00	
SRT-Right	Simple Snoring	20	6,75^a^	2,447	5,00	5,00	10,00	<0.001
	Mild OSAS	6	11,67^a^	4,082	8,75	12,50	15,00	
	Moderate OSAS	10	10,00^a^	4,714	5,00	10,00	11,25	
	Severe OSAS	27	18,59^b^	6,387	15,00	15,00	20,00	
SRT-Left	Simple Snoring	20	6,00^a^	2,616	5,00	5,00	5,00	<0.001
	Mild OSAS	6	12,50^b^	5,244	8,75	12,50	16,25	
	Moderate OSAS	10	10,00^b^	4,082	8,75	10,00	10,00	
	Severe OSAS	27	19,26^c^	5,495	15,00	20,00	20,00	
PTA-A-Right	Simple Snoring	20	9,50^a^	4,347	7,50	10,00	12,00	<0.001
	Mild OSAS	6	14,83^a^	6,494	9,25	15,00	21,00	
	Moderate OSAS	10	12,40^a^	3,307	10,00	11,50	13,75	
	Severe OSAS	27	24,15^b^	4,504	20,00	25,00	26,00	
PTA-B-Right	Simple Snoring	20	5,45^a^	4,310	,50	5,00	9,75	<0.001
	Mild OSAS	6	9,17^a^	6,210	5,00	5,50	16,00	
	Moderate OSAS	10	8,10^a^	3,381	5,00	7,50	10,25	
	Severe OSAS	27	16,85^b^	5,231	14,00	16,00	19,00	
PTA-A-Left	Simple Snoring	20	9,75^a^	4,506	6,25	10,00	12,00	<0.001
	Mild OSAS	6	21,17^b^	14,552	9,00	18,00	30,75	
	Moderate OSAS	10	16,50^b^	6,587	10,75	15,00	20,75	
	Severe OSAS	27	24,00^c^	4,515	20,00	24,00	26,00	
PTA-B-Left	Simple Snoring	20	5,10^a^	3,401	2,50	5,00	6,00	<0.001
	Mild OSAS	6	12,17^b^	7,985	5,00	9,50	21,00	
	Moderate OSAS	10	10,80^b^	6,268	5,00	9,00	15,00	
	Severe OSAS	27	15,41^b^	4,440	12,00	15,00	17,00	
SD Mean	Simple Snoring	20	98,50^a^	2,06	97,25	99,50	100,00	<0.001
	Mild OSAS	6,00	91,92^b^	3,60	88,38	92,50	95,25	
	Moderate OSAS	10,00	91,30^b^	4,05	88,25	91,75	93,75	
	Severe OSAS	27,00	87,78^c^	3,92	85,00	87,50	90,00	
SRT Mean	Simple Snoring	20	98,50^a^	2,06	97,25	99,50	100,00	<0.001
	Mild OSAS	6,00	91,92^b^	3,60	88,38	92,50	95,25	
	Moderate OSAS	10,00	91,30^b^	4,05	88,25	91,75	93,75	
	Severe OSAS	27,00	87,78^c^	3,92	85,00	87,50	90,00	
PTA-Air Mean	Simple Snoring	20	9,63^a^	4,31	7,13	10,00	12,75	<0.001
	Mild OSAS	6,00	18,00^c^	9,98	9,13	16,50	25,88	
	Moderate OSAS	10,00	14,45^b^	4,13	11,13	14,00	16,63	
	Severe OSAS	27,00	24,07^d^	3,72	21,50	23,00	26,00	
PTA-Bone Mean	Simple Snoring	20	5,28^a^	3,67	2,50	5,00	7,50	<0.001
	Mild OSAS	6,00	10,67^b^	5,96	5,00	9,75	16,13	
	Moderate OSAS	10,00	9,45^b^	4,46	5,00	8,00	13,13	
	Severe OSAS	27,00	16,13^c^	4,59	12,50	15,50	18,00	

^a^ *Fisher-Freeman-Halton exact test.* ^b^ *ANCOVA model.* ^c^ *Post-Hoc Tukey test*

**A review of** Table4 **suggested that**

-The lowest mean SD-Right, levels were seen in the severe-OSAS group, where the highest mean was seen in the simple-snoring group. The mean values in the other two groups were similar and intermediate.

- The lowest mean SD-Left value was seen in the severe-OSAS group, and there was no significant intergroup difference as regards the other three groups.
- The highest mean SRT-Right value was seen in the severe-OSAS group, where there was no significant intergroup difference between the other three groups.
- The highest mean SRT-Left value was seen in the severe-OSAS group, while the lowest mean value was from the simple-snoring group. The mean values in the other two groups was an intermediate value and there was no significant intergroup difference between them.
- The highest mean PTA-Air-Right value was seen in the severe-OSAS group, where there was no significant difference between the other three groups.
- The highest mean PTA-Bone-Right value was seen in the severe-OSAS group, where there was no significant difference between the other three groups.
- The highest mean PTA-Air-Left was seen in the severe-OSAS group, while the simple-snoring group had the lowest mean value. The mean values in the other two groups was an intermediate value and there was no significant intergroup difference between them.
- The lowest mean PTA-Bone-Left value was seen in the simple-snoring group, but there was no significant difference between the other three groups by the above mean value.

A review of the intergroup comparisons based on mean Right and Left values indicate that SD Mean and SRT Mean values were significantly higher in the simple snoring group compared to the other three groups, nevertheless, there was no significant difference between the other three groups. 

The lowest mean PTA-Air Mean value was seen in the simple-snoring group, followed by the moderate-OSAS, mild-OSAS, and severe-OSAS groups. Furthermore, the lowest PTA-Bone Mean value was seen in the simple-snoring group, followed by the moderate-OSAS and mild-OSAS groups with no intergroup differences, where the highest mean was seen in the heavy-OSAS group. 

**Fig 1:**
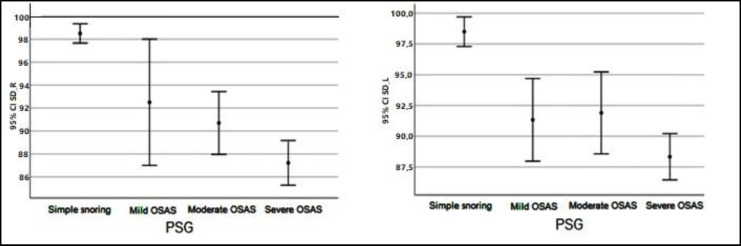
Descriptive values pertaining to SD Right and Left measurements by groups

**Fig 2:**
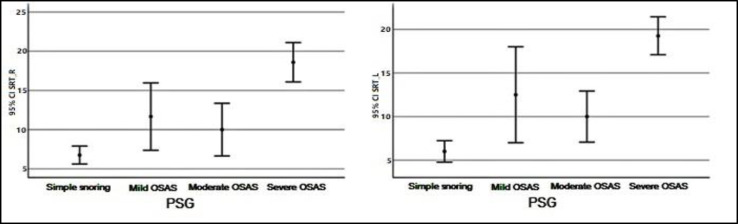
Descriptive values pertaining to SRT Right and Left measurements by groups

**Fig 3:**
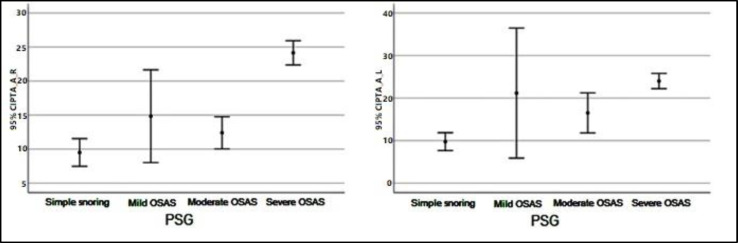
Descriptive values pertaining to PTA-Air Right and Left measurements by groups

**Fig 4:**
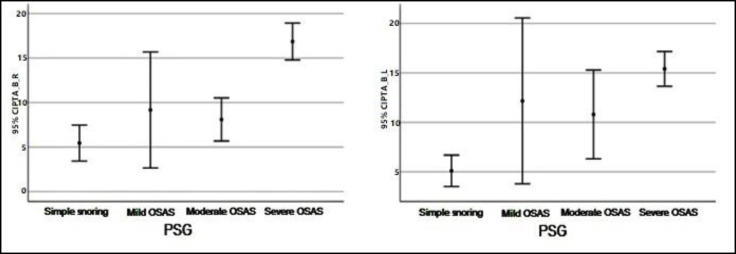
Descriptive values pertaining to PTA-Bone Right and Left measurements by groups

Descriptive values pertaining to SD Right and Left measurements by groups have shown in Figure 1. Descriptive values pertaining to SRT Right and Left measurements by groups have shown in Figure 2. Descriptive values pertaining to PTA-Air Right and Left measurements by groups have shown in Figure 3. Descriptive values pertaining to PTA-Bone Right and Left measurements by groups have shown in Figure 4.

## Discussion

Within the human body, the OSAS is well-known for its adverse effects on multiple systems, and that OSAS may further affect the vestibular and cochlear systems. Data on OSAS and the features of hearing loss were limited in previously published relevant literature. AHI is the main parameter of PSG for the diagnosis and classification of a patient with OSAS. Cochlear damage is also expected in OSAS cases given that chronic intermittent hypoxemia affects the vestibular system in patients with OSAS (11,12). A study by Kayabaşı et al. suggested a cross-sectional relationship between the severity of OSAS and increased PTT (Pure Tone Thresholds), SRT, and decreased SDS levels (13). It was suggested in previous relevant publications that the adverse effects of OSAS on the central auditory pathways might be the basic mechanism of hearing deterioration in patients with OSAS (14). On the contrary, most of the publications focusing on hearing functions in patients with OSAS reported that cochlear ischaemia owing to chronic hypoxemia and inflammation of cochlea associated with the proinflammatory phase of OSAS can cause hearing deterioration in patients (6,15).

The present study viewed the audiometric parameters associated with PSG parameters in patients with OSAS. It was not surprising that the mean PTT and SRT values were higher, and in the severe-OSAS group, the SD values were significantly lower. The present study suggests the conclusion that patients with moderate to severe OSAS might be at risk of hearing deterioration independent of aging process. In the present study, severe-OSAS had significant effects on all the hearing functions. Nevertheless, the fact that patients’ BMI levels were not included in the analyses is a limitation of the study. A review of the relevant literature suggested that the adverse effects of OSAS on tissue oxygenation and vascular function were comprehensively studied. Hypoxemia-associated consequent vasoconstriction and chemoreflex stimulation, which were sustained even during the normoxic daytime wakefulness in patients with OSAS, were previously documented (16,17). 

The authors suggested in the latest study that “obstructive sleep apnea syndrome did not affect cortical processing of auditory afferent stimuli during sleep”(16,18). Cochlear cells may be harmed by recurrent ischemic damage due to apneic crisis (1,5). 

The results of the present study showed that patients with OSAS with an index of apnea-hypopnea level of less than 15 did not have any auditory dysfunction. Nevertheless, a mild sensorineural hearing loss was observed in patients with both moderate and severe obstructive sleep apnea syndrome.

## Conclusion

The results of the study suggested that chronic hypoxemia due to obstructive sleep apnea syndrome might have adverse effects on both the speech discrimination and hearing.
